# Touch in psychotherapy: Experiences, desires and attitudes in a large population survey

**DOI:** 10.1080/10503307.2025.2534971

**Published:** 2025-07-25

**Authors:** Michal Tanzer, Athanasios Koukoutsakis, Ilia Galouzidi, Paul M. Jenkinson, Claudia Hammond, Michael J. Banissy, Aikaterini Fotopoulou

**Affiliations:** 1 Research Department of Clinical, Educational and Health Psychology, University College London, UK; 2 Faculty of Psychology, Psychotherapy and Counselling, The Cairnmillar Institute, Australia; 3 Department of Psychology, University of Sussex, UK; 4 Department of Psychology, Goldsmiths, University of London, UK; 5 School of Psychological Science, University of Bristol, UK

**Keywords:** therapeutic touch, attachment style

## Abstract

**Objective:**

Social touch has known therapeutic effects on physical and mental health. However, research on the role of touch in psychotherapy is limited. The frequency of touch in psychotherapy, and the individual differences that influence clients’ perceived quality and desirability of touch, remain unknown.

**Methods:**

We utilized the largest-to-date UK survey on touch, identifying 6878 individuals who reported receiving psychological therapy in the last 10 years, to explore: (1) the perceived quantity and affective quality (positive/negative) of therapeutic touch experiences, (2) the desirability of touch, and (3) key traits such as attachment style that may influence the perceived meaning and desirability of touch.

**Results:**

Touch was reported by 30% of responders, with 70% of them stating it communicated support. Importantly, 4% reported it as inappropriate, and 5% rated their experiences as negative. Additionally, 40% of responders expressed some desire to be touched by their therapist. Higher scores on avoidant attachment were negatively associated with positive ratings of touch. The desire to be touched was associated with attachment style, with individuals scoring higher on anxious attachment reporting a greater desire for touch.

**Conclusion:**

This exploratory survey highlights the need for further investigation into the potential benefits and risks of touch interventions.

## Introduction

### Social-Touch in Psychotherapy

Touch in psychotherapy, defined as intentional, ethical, and non-erotic physical contact initiated by the therapist, has been associated with numerous positive therapeutic outcomes, including mental and physical health benefits (Berendsen, 2017; Horton et al., 1995; Hunter & Struve, 1998; Myers et al., 2022; Peloquin, 1989; Phelan, 2009; Salzmann-Erikson & Eriksson, 2005; Strozier et al., 2003). Touch interventions, such as mindfulness combined with active touch and rhythmic calming touch have been shown to reduce symptoms of depression (Stötter et al., 2013) grief (Kempson, 2000), and dementia (Woods et al., 2005). Touch within the therapeutic relationship has also been shown to strengthen the therapeutic alliance (Myers et al., 2022), while its absence was identified as a significant barrier to effective grief management during the COVID-19 pandemic (Scheinfeld et al., 2021). Touch within the therapeutic relationship has also been shown to strengthen the therapeutic alliance (Myers et al., 2022), while the absence of touch was identified as a significant barrier to effective grief management during the COVID-19 pandemic (Scheinfeld et al., 2021).

This study, focused specifically on non-transactional, emotionally meaningful forms of touch in psychotherapy (e.g., hugging, holding, and stroking), excluding incidental or purely procedural contact (e.g., handshakes or accidental brushing). Touch in psychotherapy is theorized to convey a variety of meanings, including reassurance, grounding (Eyckmans, 2009), legitimation, and acceptance (Mintz, 1969), as well as a symbolic representation of parental care (Durana, 1998), and the differentiation and awareness of self—other boundaries (Fotopoulou & Tsakiris, 2017; Leder & Krucoff, 2008; Westland, 2011). However, empirical support for the benefits of touch in psychotherapy, particularly from the client’s perspective, remains limited, with much of the literature relying on theoretical exploration, psychotherapists’ experiences, relatively small samples or case studies (Davis et al., 2017; Geib, 1998; Horton et al., 1995; Hunter & Struve, 1998; Schlesinger & Appelbaum, 2000; Westland, 2011), restricting the breadth of conclusions that can be drawn.

Surveys on the use of touch in psychotherapy suggest most psychotherapists have employed touch at least once (Pope et al., 1987; Strozier et al., 2003) but see (Stenzel & Rupert, 2004; Strozier et al., 2003). For example, a survey of 666 psychotherapists found that nearly half of the respondents believed non-erotic contact behaviours (specified as hugging, kissing, or affectionate touch) could benefit clients in specific situations, such as during acute distress, to provide emotional support, when working with younger clients, or with clients who have a history of neglect (Holroyd & Brodsky, 1977). However, attitudes toward touch can vary depending on therapeutic orientation (Holroyd & Brodsky, 1977). Among the surveyed therapists, 68% of those with a psychodynamic orientation and fewer than 20% of humanistic therapists believed that non-erotic touch should not be used, as it unlikely to help clients. Therapists practising psychodynamic therapy or cognitive–behavioural therapy (CBT) were the least likely to use these touch interventions (Holroyd & Brodsky, 1977). In CBT, touch might occasionally be used as part of a specific intervention, such as grounding techniques to reduce anxiety, while in psychodynamic therapy, a gentle pat on the shoulder might be offered to convey emotional support. By contrast, therapists with a humanistic approach were the most likely to incorporate touch (Holroyd & Brodsky, 1977). These findings align with the emphasis on somatic therapy integration seen in body-oriented therapies, such as bioenergetics or biodynamic therapies (Lowen, 1976), somatic (Caldwell, 1997), and Gestalt approaches (Perls, 1973; Rogers, 1970), which regard touch interventions (for example, a light touch on the client’s back as a way to re-engage with their emotions) as an acceptable intervention for addressing both psychological and physical well-being (see Phelan, 2009).

From the client’s perspective, evidence is also limited. In one study, 69% of 159 psychotherapy clients reported that touch fostered a sense of connection and enhanced feelings of closeness with their therapist (Horton et al., 1995). These positive perspectives on touch in psychotherapy stand in contrast to some concerns about its potential negative effects (Gabbard, 2017; Geib, 1998; Pope & Bouhoutsos, 1986). For example, in a study by Horton et al. (1995), ten respondents out of 159 attributed negative meanings to touch interventions in therapy, with six participants mentioned the therapist’s discomfort and four feeling that the touch did not meet their needs (Horton et al., 1995). Clients may also perceive touch as benefiting the therapist more than themselves or as undermining their sense of control (Geib, 1998). Such findings underscore the importance of a nuanced approach to touch, one that considers the diverse factors influencing its reception and interpretation.

The impact of touch in psychotherapy is shaped not only by its potential benefits or risks, but also by the personal characteristics and cultural backgrounds of both therapists and clients. Gender, for instance, plays a significant role, as women generally report finding touch more comforting and acceptable than men - a dynamic likely reflected in therapeutic settings (Stenzel & Rupert, 2004; Suvilehto et al., 2015). In Western societies, affectionate touch, such as hugging or holding hands, is often perceived as more acceptable between women than between men, with women deriving greater comfort and emotional satisfaction from it (Suvilehto et al., 2015). Cross-cultural research further highlights variations in touch perception. For example, although no differences were observed between women in the UK and South Africa in terms of self-reported general attitudes toward touch, South African women rated certain types of experimentally induced touch as more pleasant than women in the UK. In addition, a study comparing participants from the UK and Japan found that UK participants experienced social touch as more pleasant than their Japanese counterparts (Suvilehto et al., 2019, 9).

While the aforementioned surveys on touch interventions in psychotherapy were relatively common, most were conducted two to five decades ago. This temporal gap raises concerns about their applicability to current psychotherapy settings, as ethical standards, cultural attitudes and professional guidelines have since evolved significantly. Movements such as #MeToo, alongside broader public dialogue on sexual misconduct and harassment, have reshaped how past unwanted sexual contact experiences are now recognized as sexual assault rather than ambiguous or minimized encounters (Cary et al., 2022; Jaffe et al., 2021; Westland, 2011). Within psychotherapy, this shift has coincided with a stronger focus on ethical practice, risk management and litigation concerns, which may deter open discussion about rare appropriate uses of touch (Cary et al., 2022; Harrison et al., 2012; Jaffe et al., 2021; Stenzel & Rupert, 2004; Westland, 2011).

Non-body oriented psychotherapy training programmes and professional guidelines such as those issued by the American Psychologist Association or the British Association for Counselling and Psychotherapy often do not explicitly mention touch interventions, and when they do, it is typically framed in terms of ethical risk and boundary violations (Phelan, 2009; Zur & Nordmarken, 2011). As a result, trainees often receive little practical guidance beyond cautionary messages, reinforcing a predominantly avoidant stance (Zur, 2007; Zur & Nordmarken, 2011). In contrast, some somatically oriented programmes, such as Somatic Experiencing, include structured training in touch interventions (e.g., hand on a shoulder) to support emotion regulation or educing anxiety symptoms (Kuhfuß et al., 2021). These trainings emphasize context, ethical awareness, consent, and clinical skill in the application of touch. Such contrasting approaches highlight the lack of consensus and the need for clearer, systematic reviews and empirically-based guidelines on the appropriate use - or avoidance - of touch in therapy (Bonitz, 2008; Phelan, 2009; Westland, 2011).

### From Social Touch to Developmental Influences

Attitudes toward touch are also shaped by early touch experiences, which contribute to the formulation and maintenance of attachment style, social affiliations and social bonds, including dimensions of adult attachment style (Ainsworth, 1979; Bowlby, 1969; Brauer et al., 2016; Dunbar, 2010; Fotopoulou & Tsakiris, 2017; Montagu, 1978; Morrison, 2016; Reite, 1990; Shaver & Mikulincer, 2010; Takeuchi et al., 2010). For example, higher frequencies of benevolent parental touch during childhood are associated with a positive perception of current romantic partners and reduced depressive symptoms in young adults (Takeuchi et al., 2010). Conversely, individuals with a high avoidant attachment style report less frequent touch in romantic relationships (Debrot et al., 2021), derive less enjoyment from cuddling with partners or their children (Chopik et al., 2014) and tend to distance themselves from others (Shaver & Mikulincer, 2010). Similarly, avoidant attachment scores have also been found to moderate the relationship between received touch and state security, with higher scores on this style correlating with diminished benefits from touch experiences (Jakubiak & Feeney, 2016). Remarkably, scores in anxious attachment style did not exhibit the same moderation effect or preferences for touch (Chopik et al., 2014; Jakubiak & Feeney, 2016). This pattern aligns with the notion that individuals high on anxious attachment style approach intimacy in an ambiguous manner due to fears of rejection (Ainsworth, 1989). While these individuals may desire touch, they may simultaneously resist it (Jakubiak & Feeney, 2016).

Laboratory studies have further demonstrated that the same touch (e.g., stroke on the hand) can feel compassionate to one person and threatening to another, depending on attachment style (Krahé et al., 2018). Despite growing evidence of therapeutic effects of interpersonal touch throughout the lifespan, limited and conflicting empirical data regarding the prevalence and affective quality of touch in psychotherapy. This gap—another focus of the current study—needs to be addressed for advancing understanding in this field.

### The Present Study

This preregistered study involved a sample of individuals from the United Kingdom who participated in a large-scale national survey on touch. Participants who had undergone psychotherapy in the past 10 years were asked about their experiences with touch in therapy.

Our preregistered *main* hypotheses (see also OSF: https://osf.io/jr4vc) explored various interrelated dimensions of touch in psychotherapy—specifically, the desire for touch, the quantity of touch experiences, and their affective quality (both in general and in relation to the communication it may convey) from the clients’ perspective. Additionally, and as preregistered, we investigated these perceptions across different subgroups, comparing talking therapies to body-oriented psychotherapy (see also Therapy Uptake, Duration and Modality).

### Hypotheses Regrading Desire for Touch

(H1a) We hypothesized that the majority of individuals who have undergone talking therapy (e.g., counselling, psychotherapy, CBT or other talking cure), regardless of whether they have experienced touch in their therapy, would report a desire for touch (Geib, 1998; Horton et al., 1995; Hunter & Struve, 1998; Schlesinger & Appelbaum, 2000).

(H1b) We expected that longer therapy durations, often associated with the exploration of developmental issues (Juul et al., 2019) and increased feelings of closeness and intimacy between clients and therapists (Horton et al., 1995), would result in a greater desire for touch.

(H1c) We hypothesized that the desire for touch would be associated with the client’s attachment style dimension. Specifically, we expected that individuals with higher scores in avoidant attachment style would report on lower (Ainsworth, 1989; Shaver & Mikulincer, 2010), while those higher scores in anxious attachment style would show a greater desire for touch, driven by their need for closeness and reassurance (Jakubiak & Feeney, 2016).

(H1d) We expected that individuals with a more positive attitude toward touch would express greater desire for touch in therapy, as they would be less likely to avoid touch-related activities (Turp, 2000).

### Hypotheses regarding general affective quality of touch experiences

(H2a) Among those who *had experienced* touch in therapy, we predicted that lower scores on anxious or avoidant attachment style dimensions would be positively associated with more positive ratings of their touch experiences.

(H2b) We expected that individuals with higher scores on positive developmental touch history would rate their therapeutic touch experiences more positively (Takeuchi et al., 2010).

### Hypotheses regarding specific aspects of affective quality of touch experiences

(H3a) Among those who *had experienced* touch in therapy, we predicted that for most individuals, touch would be seen as supportive. However, we also anticipated that some participants would report these experiences as inappropriate (Geib, 1998; Horton et al., 1995).

(H3b) We expected that these different perceptions of psychotherapeutic touch experiences would be moderated by attachment style, developmental touch history and general touch attitudes.

Lastly, (H4) the study also explored the quantity and quality of touch experiences across different psychotherapy modalities, such as talking therapies versus body-oriented psychotherapy. Detailed hypotheses related to these aspects are available in our preregistration (OSF: https://osf.io/jr4vc).

## Materials and Methods

### Procedure

The survey commenced on January 21st, 2020, and concluded on March 30th, 2020. It was conducted on an online platform (touchtest.org) hosted by Goldsmiths, University of London, and launched via BBC Radio 4. The survey link was disseminated through radio broadcasts, associated websites, and social media channels, both via individual and institutional accounts. Participants were provided with an information page detailing the study’s purpose were required to provide informed written consent to participate. This study is part of a broader survey exploring individuals’ attitudes toward the physical experience of touch and its relationship to health and well-being. The current survey specifically focused on the optional psychotherapy section, where participants were asked whether they had undergone psychological therapy in the last 10 years.

Questionnaires were presented in random order, with no time limit for completion. Participants could pause and return to the survey as often as they wished within a seven-day period (From the whole survey 15,449 participants (90%) completed the survey on the same day, while 2326 participants took more than one day to finish). They were also free to withdraw from the study at any point. The study received ethical approval from the Research Ethics and Integrity Sub-Committee, Goldsmiths, University of London, (Project reference 1521).

### Participants

A total of 39,254 participants completed the survey. Of these 19,425 adults (aged 18 and over) from the UK chose to respond to the optional psychotherapy section (14,421 women; 4724 men, 201 non-binary or preferring to self-describe, and 79 participants who did not disclose their gender).

For the sample of interest, the following preregistered inclusion criteria were applied: (1) Individuals with demographic characteristics that constituted less than 1% of respondents were excluded. (2) Participants who completed fewer than 80% of items on any given scale or subscale were excluded. Additionally, individuals who did not respond to demographic questions (used as control variables) were removed. After these exclusions, the final sample comprised 17,775 participants, including 13,384 women and 4321 men. Of this sample, 39% (*n* = 6878; 5547 women and 1331 men, see Table 1 for detailed demographics) reported having undergone psychological therapy in the past 10 years. It is also worth noting that, upon reviewing the demographics of our sample, we identified a disproportionately high number of respondents who listed their age as 18 (*n* = 147; see Figure 1 in Supplementary Materials). Given that participation required individuals to be older than 18 years old, we were concerned that this overrepresentation might reflect misreporting by younger respondents (e.g., Ward & Meade, 2023). To maintain data integrity and adhere to our eligibility criteria, we excluded all responses from individuals who reported being 18 years old, thereby minimizing any potential bias or skewing of the data.

### Instruments

The variables and scoring methods described in this section were preregistered and followed, unless otherwise specified. Due to time constraints and the aim to ensure the survey’s broad appeal to the public, we utilized a limited number of questions and response options.

#### Demographic questions

Participants provided anonymous information on age, gender, sexuality, ethnicity and religion. This information was used to define the final sample as detailed earlier and served as control variables in the analyzes (see Table 1).

#### Therapy uptake, duration and modality

We measured Therapy Uptake with the question: “Have you been in psychological therapy at any time in the past 10 years?”. Participants selected from three response options: “Yes”, “Prefer Not to Say” and “No”. For participants who answered “Yes” therapy duration was assessed with the questions: “For how long in total?” Response options included “Less Than 3 Months”, “3 Months to 1 Year” and “More Than 1 Year”.

The survey also included a question about the therapeutic modality: “What type of therapy did you take part in?” Response options included: “Psychotherapy”, “Counselling”, “Cognitive–Behavioural Therapy (CBT)”, “Talking Therapy” and “Other. Please specify”. While not ideal, we were encouraged by some of the “research users” (e.g., the BBC Radio representative) to include both broad and not-mutually exclusive categories (e.g., psychotherapy, counselling, talking therapy), as well as specific modality, such as CBT. These terms were considered the most familiar to the public, and the aim of this question was to help participants recognize their own therapeutic experience rather than to differentiate between these terms.

For the primary analysis, responses for “Psychotherapy”, “Counselling,” “CBT”, and “Talking Therapy” were aggregated into a single category labelled “talking therapies.” This approach reflects the non-mutually exclusive nature of these options, as CBT is both a form of psychotherapy and a talking therapy. Any supplementary analyzes examining CBT separately from other therapies have also been reported for completeness but are designated as exploratory throughout.

In a separate question, participants were asked to specify whether their therapy included body-oriented aspects such as biodynamic or massage therapy, as opposed to predominantly talking therapy. Four response options were provided: “Yes”, “No”, “Not sure”, Prefer “Not to Say”. This information was used to categorize therapies into “body-oriented” vs “non-body-oriented therapies” groups for subsequent analyzes.

#### Desire for touch

Participants’ desire for physical touch from their therapist was assessed with a single item: “Have you ever wanted your therapist to hug, hold, or touch you in some way?” Responses were recorded on a 7-point scale ranging from 1 (Never) to 7 (Always). Frequency distributions of responses are provided in Table 1S.

#### Touch experiences. Quantity of touch experiences with therapists

This was measured using a single item: “Have you ever experienced some sort of physical contact with your therapist beyond accidental contact or a formal handshake?” Responses were recorded using four options: “Never”, “Once”, “Less Than 10” and “More Than 10”.

#### Overall affective quality of touch experiences

This was assessed using the average score of three items: (1) “Overall, how do you regard the physical contact which occurred in your therapy”? (2) “How were the feelings about yourself affected by the touch?” (3) “How were your feelings about your therapist affected by the touch?” Participants rated each item from 1 (Very Negative) to 7 (Very Positive). *Supportive and Bonding Experiences* assessed using a single item: “To what degree did you feel the touch communicated acceptance or support and enhanced your connection, or bond with the therapist?” and *Intrusive and Inappropriate Experiences* were assessed using a different item: “To what degree did you feel the touch violated the boundaries of the therapeutic relationship and was inappropriate?”. Both items rated from 1 (Never) to 7 (Every Time). Frequency responses can be found in Table 1S.

#### Adult attachment style dimensions

Adult attachment style was assessed using the Experiences in Close Relationships-Short Form (ECR-S; Wei et al., 2007), a 12-item self-report dimensional measure designed to evaluate avoidant and anxious attachment styles. The ECR-S adopts a dimensional approach, yielding continuous scores for each dimension. The scale includes six items for avoidant attachment and six for anxious attachment, with responses rated on a 7-point Likert scale ranging from 1 (Strongly Disagree) to 7 (Strongly Agree). Scores for each dimension were averaged, with higher scores indicating higher levels of insecure attachment. The ECR-S has been extensively validated (Wei et al., 2007) and demonstrated good internal consistency in this study: Cronbach’s *α* = .86 for avoidant and *α* = .85 for anxious attachment.

#### Attitudes to touch

General attitudes towards touch were assessed using a composite score derived from two questionnaires: the Social Touch Questionnaire (STQ; Wilhelm et al., 2001) and the Touch Experiences and Attitudes Questionnaire (TEAQ; Trotter et al., 2018). An exploratory and confirmatory factor analysis conducted specifically for this survey (Bowling et al., 2024), identified a six-factor structure, which was then used to address specific hypotheses (see below). The six factors identified were: “Dislike of Physical Touch”, “Childhood Touch/ Developmental Touch History”, “Attitudes to Intimate Touch”, “Current Intimate Touch”, “Liking of Physical Touch” and “Attitudes to Self-Care”. See supplementary materials for more details on these instruments.

#### Developmental touch history

The assessment of developmental touch history, represented the factor “Childhood Touch”, was based on a composite average score. This score was derived from two items on the TEAQ related to positive touch experiences in childhood (“My parents were not very physically affectionate towards me during my childhood”, and “As a child my parents would tuck me up in bed every night and give me a hug and a kiss goodnight”) and one item from the STQ (“As a child, I was often cuddled by family members (e.g. parents, siblings))”. Cronbach’s *α* = .86.

### Data Analysis

We first conducted frequency analyzes for each dependent measure. For continuous dependent variables, such as overall attitudes towards touch in therapy, we employed separate multilevel models (MLM). This approach was specified in our pre-registration to account for potential variance explained by group effects of categorical variables, including ethnicity, sexuality, gender, and religion. However, in practice we found that these effects were minimal (interclass correlation coefficient (ICC) <0.001), suggesting that they did not contribute meaningfully to the results and were not interpreted further. In all MLM analyzes, we controlled for these categorical variables as random effects, but we kept these variables in the final models only wherever ICC exceeded 0.001.

For hypotheses involving categorical dependent variables, such as the type of psychotherapy or dichotomized responses (e.g., “Never” or “Almost Never” vs “Rest” in desire for touch), we used MLM Binomial Logistic regression. This choice was based on the pre-registered assumption that responses would not be normally distributed but would instead split into two poles. However, we also report the results of MLM treating the dependent variable as a continuous, for comparison,

In all analyzes, independent variables (e.g., type or duration of therapy, attachment style dimensions or attitudes to touch), were entered as fixed effects of interest (See Table 2S for intercorrelations between independent variables). Normality checks were conducted for continuous dependent variables, and Principal Component Analysis (PCA) was preformed to address potential multicollinearity between independent variables. Given the pre-registered hypotheses and the substantial sample size (ranging from 1596 to 6768), we used a conservative alpha value of *p* < 0.01 and marginal *R*^2^ ≥ 0.01.

## Results

We begin by characterizing the sample to provide context for subsequent analyzes. Each section follows the order of our preregistered hypotheses. First, we report the frequency of responses, followed by hypothesis testing. The subsequent sections are structured as follows: (H1) hypotheses regarding touch desire, (H2) hypotheses addressing the general affective quality of touch experiences, (H3) hypotheses focusing on specific aspects of the affective quality of touch experiences, and (H4) hypotheses examining the quantity and quality of touch experiences across different psychotherapy modalities, such as talking therapies versus body-oriented psychotherapy.

### Sample Characteristics

A total of 6878 individuals (5547 women and 1331 men; *M*_*Age*_ = 56.1 ± 14.63) reported having received psychological therapy in the past 10 years (see Participants for the full survey sample and Table 1 for demographics of the “psychotherapy” group). Among them 89% (*n* = 6129; women = 4947; men = 1182) reported participating in non-body-oriented therapies, 8% (*n* = 570; women = 459; men: 111) indicated that their treatment was body-oriented, and 3% (*n* = 181; women = 148, men = 33) were unsure or preferred not to say.

### Desire for Touch in Relation to Therapy Group, Duration, Attachment and Touch Attitudes

**Figure 1:**
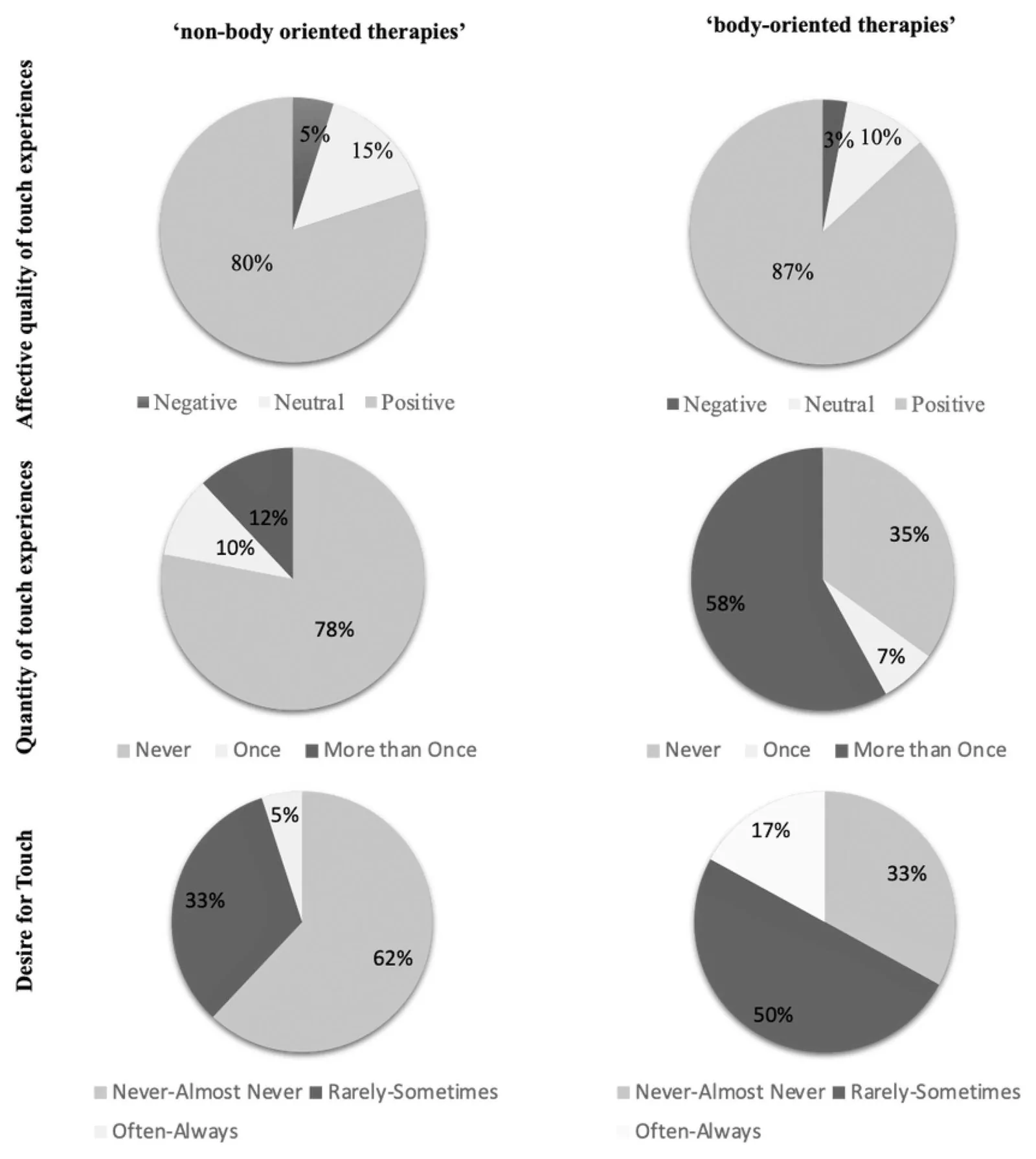
Percentages of individuals to report on touch experiences in “non-body-oriented therapies” and “body-oriented therapies”.

Within the entire *psychotherapy* sample, 60% of participants reported “Never” or “Almost Never” wanting their therapist to hug, hold or touch them, while 40% reported “Rarely” to “Always”, indicating some level of desire for touch (for a detailed breakdown by therapy modality, see Figure 1 and Table 1S).

To test H1a, which predicted that, within the *talking therapies* group, the number of individuals who reported on some desire for touch will be significantly larger than those who never have had desire, we conducted a logistic MLM. Desire for touch was treated as a dichotomous dependent variable (“Never” or “Almost Never” vs. “Rarely” to “Always”), with therapy duration, attachment style dimension (avoidant, anxious) and general touch attitudes (total summed score of the TEAQ and STQ) as the independent variables $\left(R_{Marginal\;}^{2}=0.15;n=4816\right)$.

H1a was not supported, within the *talking therapies group*, there was no significant difference in the odds of individuals never wanting touch compared to those desiring some touch (*OR* = 0.86, *p* = .40 95% CI [0.60;1.23]). Supporting H1b, longer therapy duration within the *talking therapies group* was associated with a greater desire for (*OR* = 2.00, *p* < .001, 95%CI [1.79;2.25]). Age was also positively associated with a greater desire for touch (*OR* = 1.01, *p* = .001, 95%CI [1.00;1.01]). Supporting H1c and H1d, higher anxious attachment scores and more *positive* touch attitudes significantly increased the likelihood of desiring touch (*OR* = 1.35, *p* < .001, 95%CI [1.26;1.43]; *OR* = 1.82, *p* < .001, 95% CI[1.71;1.95]) respectively. However, contrary to H1c, higher avoidant attachment scores did not significantly reduce the odds of reporting a desire for touch (*OR* = 0.99, *p* = .69, 95%CI [0.93;1.05]). The same significant effects emerged when the dependent variable was treated as a continuous rather than binary (see Supplementary Table 3S).

Finally, in a separate pre-registered analysis (as outlined in the pre-registration), we compared the two therapy groups (body-oriented vs. non-body-oriented). Results indicated that participants who have had *body-oriented therapy* had significantly higher odds of reporting a desire for touch compared to those in *non-body-oriented therapies* $(R_{Marginal\;}^{2}=0.04;\;n=6686;\;OR=2.80,\;p=\;<.001,\;95\%\text{CI}\;[2.29;\;3.41])\;$. We also conducted an exploratory analysis to examine whether participants with different attachment styles (anxious or avoidant) were more likely to engage in body-oriented therapy, however found no significant associations between these measures (see Supplementary Materials)

### Affective Quality of Touch Experiences in Relation to Developmental Touch History, Attachment, and Attitudes to Touch

Amongst participants who reported experiencing touch in therapy at least once (*n* = 1689), the majority (81%, *n* = 1374) rated the experience as positive, while 14% (*n* = 240) rated it as neutral, and 5% (*n* = 75) rated it as negative (Figure 1 and Table 1S for frequencies by therapy group).

To test our predictions (H2), we conducted MLM analysis examining whether lower scores on anxious attachment or avoidant attachment, as well as more positive attitudes toward touch, were associated with more positive evaluations of touch experiences in therapy. Ratings of the *affective quality of touch experiences* served as continuous dependent variable, while *therapy group* (body-oriented vs. non-body-oriented therapies), was included as a categorical independent variable, alongside continuous measures of attachment style dimension and attitudes toward touch $\left(R_{Marginal\;}^{2}=0.1;n=1610\right)$.

Supporting H2a, higher avoidant attachment scores were significantly associated with less positive ratings of touch experiences (*b* = −0.11, *p* < .001, 95%CI [−0.16;−0.06]). However, anxious attachment scores were not significantly associated to the affective quality of touch experiences (*b* = −0.02, *p* = .54, 95%CI [−0.07;0.04]).

Additionally, three factors from the combined TEAQ_STQ measure—*attitudes to intimate touch, liking of physical touch* and *attitudes to self-care*—were positively associated with the affective quality of touch experiences in therapy (*b* = 0.12, *p* < .001, 95%CI [0.06;0.17]; *b* = 0.26, 95%CI [0.21;0.32], *p* < 0.001; *b* = 0,08 *p* = 0.004, 95%CI [0.03;0.14], respectively). This indicates that individuals who scored higher on these measured reported more positive touch experiences. *Dislike of physical touch* was negatively associated with the affective quality of touch experiences (*b* = −.0.23, *p* < .001, 95%CI [−0.28;−0.18]), suggesting that individuals with higher scores on this measure rated their touch experiences less positively. We found no evidence supporting H2b, as *developmental touch history* and *current intimate touch* were not significantly associated with the affective quality of touch experiences in therapy (*b* = −0.01, *p* = .64, 95%CI [−0.06;0.04]; *b* = −0.02, *p* = .41, 95%CI [−0.07;0.03]). Additionally, *therapy group* was not significantly associated with the affective quality of touch experiences (*b* = 0.18, *p* = 0.50, 95%CI [−0.35;0.70]; see Table 4S).

### Inappropriate or Supportive Experiences of Touch in Relation to Developmental Touch History, Attachment and Attitudes to Touch

Among individuals who reported experiencing touch in therapy at least once, 88% (*n* = 1489) reported that the experience was “Never” or “Almost Never” inappropriate, while 3% (*n* = 38) reported that it was “Almost Every Time” or “Every Time” inappropriate. Additionally, 65% indicated that touch was “Always” or “Almost Always” supportive and enhanced the therapeutic bond with the therapist, whereas 4% (*n* = 79) reported that it was “Never” or “Almost Never” supportive and enhanced the therapeutic bond (Figure 1).

To examine the relationship between attachment style dimensions, touch attitudes, and the perception of touch as inappropriate, we conducted a logistic MLM $\left(R_{Marginal\;}^{2}=0.07,\;n=1685\right)$. Attachment style dimensions and touch attitudes were included as independent variables, while touch as inappropriate served as a dichotomous dependent variable (“Never” or “Almost Never” vs. “Infrequently” to “Every Time”). We controlled for the reported amount of touch in therapy. As hypothesized (H3a), within the subgroup of individuals who experienced touch in therapy, the odds of perceiving it as a violation of treatment boundaries or inappropriate were significantly smaller than the odds of not perceiving it as such (*OR* = 0.08, *p* < .001, 95% CI [0.03;0.20]). Moreover, positive touch attitudes (*liking of physical touch*) were associated with reduced odds of perceiving touch as inappropriate (*OR* = 0.79, *p* = .002, 95%CI [0.68;0.91]), whereas *disliking physical touch* was associated with increased odds of perceiving touch as inappropriate (*OR* = 1.50, *p* < .001, 95%CI [1.28;1.76]).

Higher anxious attachment scores were associated with an increased likelihood perceiving touch as inappropriate (*OR* = 1.25, *p* = 0.006, 95% CI[1.07;1.46]). To further explore these effects, we conducted a linear regression, treating the dependent variable as a continuous measure. In this analysis, higher scores on both attachment style dimensions were positively associated with higher ratings of touch as inappropriate, though avoidant attachment scores only showed a trend (*p* = 0.056; see Table 5S).

To examine the relationships between attachment style dimensions, touch attitudes, therapy group and perceptions of touch as communicating “acceptance or support and to enhance the therapeutic alliance”, we again used logistic MLM $(R_{Marginal\;}^{2}=0.08;\;n=1609)$. In this model, perceived supportiveness of touch served as a dichotomous dependent variable (“Never” or “Almost Never” vs. “Infrequently” to “Every Time”). Attachment style dimensions, touch attitudes and therapy group (body-oriented vs. non-body-oriented), were included as independent variables, while we controlled for the reported amount of touch in therapy. We found no significant interaction with therapy group (*OR* = 0.92, *p* = .68, 95% CI [0.62;1.36]), suggesting that the type of therapy (body-oriented vs. non-body-oriented) was not associated with increased odds of perceiving touch as supportive or as communicating acceptance.

In addition, as hypothesized (H3b), a positive attitude toward touch (*liking of physical touch*) was associated with increased odds of perceiving therapeutic touch as supportive (*OR* = 1.48, *p* < .001, 95%CI [1.30;1.69]). Conversely, *disliking of physical touch* was linked to decreased odds of perceiving touch as supportive (*OR* = 0.71, *p* < .001, 95%CI [0.61;0.81]). Attachment style dimensions and developmental touch history were not significantly associated with these odds (anxious: *OR* = 1.01, *p* = .92, 95%CI [0.88;1.16]; avoidant: *OR* = 0.90, *p* = .011, 95%CI [0.78;1.03]; developmental touch history: *OR* = 1.05, *p* = .54, 95%CI [0.91;1.21]). Similar results emerged when these effects were explored using linear regression.

### Quantity of Touch Experiences in Relation to Therapy Type and Duration

Among individuals receiving predominantly *talking therapies*, 75% reported never experiencing physical contact with their therapist (beyond accidental contact or a formal handshake), while 21% reported experiencing touch at least once (see Figure 1; Table 1S). In contrast, among those receiving *body-oriented therapy*, 34% reported never experiencing physical contact with their therapist, while 65% reported experiencing touch at least once (see Figure 1 and Table 1S).

To compare the two therapy groups (body-oriented vs. non-body-oriented), we used linear MLM, with the amount of touch as an ordinal dependent variable. As expected, individuals in the *body-oriented therapy* group reported significantly more touch than those in *non-body-oriented therapies* (*b* = 1.16, *p* < .001, 95% CI [1.09;1.23]; $R_{Marginal\;}^{2}=0.14;\;n=6683$.

Next, to test our pre-registered prediction that therapy duration would be positively associated with the amount of reported touch—particularly in the *body-oriented therapy group*—we conducted another linear MLM $\left(R_{Marginal\;}^{2}=0.22;n=6677\right)$. The quantity of touch was the ordinal dependent variable, while therapy duration, therapy group, and their interaction served as independent variables. As expected, we found a significant interaction between therapy group and therapy duration (*b* = 0.19, *p* < .001, 95%CI [0.08;0.31]), indicating that while therapy duration was associated with an increase in the amount of touch in the *non-body-oriented therapies* (*b* = 0.38, *p* < .001, 95%CI [0.34;0.41]), this relationship was even stronger in *body-oriented therapy*.

## Discussion

This study, using data from the UK’s largest touch survey to date, addressed significant gaps in the literature by examining clients’ experiences and attitudes toward touch in psychotherapy.

Interestingly, although most respondents (89%) reported that their psychotherapy did not include body-focused aspects, and 75% had never experienced physical contact with their therapist, 40% still expressed some desire for touch. Among those who had experienced touch in therapy, most rated it positively (81%), with 65% perceiving it as supportive and enhancing the therapeutic bond. Moreover, 88% did not find their touch experiences inappropriate. Importantly, 3-5% of the respondents reported negative and inappropriate touch experiences with their therapists, highlighting the need for caution in clinical practice.

Our first hypothesis which examined the relationship between touch desire and body-oriented therapy, was confirmed. Individuals undergoing *body-oriented therapy* reported a significantly greater desire for touch compared to those in *talking therapies*. This finding aligns with the nature of body-oriented therapies, which often incorporate physical contact and focus on therapeutic touch interventions (e.g., Changaris, 2019). It is thus unsurprising that those who choose such therapies tend to have a greater desire for touch. Additionally, within the *talking therapies* group, as predicted, longer therapy duration was associated with a greater desire for touch, possibly reflecting an evolving therapeutic bond over time (McParlin et al., 2022; Tschuschke et al., 2020), or differences in the nature of issues addressed in short-term versus long-term therapy (see Juul et al., 2019). However, our broad categorization of therapy duration limits our ability to detect non-linear or dose– response effects (Robinson et al., 2019), Future studies could benefit from investigating these dynamics using session-by-session analyzes of touch perceptions (Atzil-Slonim et al., 2023), which would offer deeper insights into how therapy length and touch interact.

Interestingly, in line with our hypothesis, we found that individual differences in attachment style dimensions and current attitudes toward touch were associated with the desire for touch. Specifically, individuals with higher anxious attachment scores and more positive touch attitudes reported greater desire for touch, while those with higher avoidant attachment scores reported less. Anxious attachment, often characterized by a persistent need for reassurance and proximity (Shaver et al., 2005), may explain why individuals with this attachment style exhibit a greater desire for touch. Relatedly, avoidant attachment, which involves a tendency to distance oneself emotionally and cope alone (Shaver & Mikulincer, 2010), likely underpins the reduced willingness to engage with therapeutic touch in this group.

To our knowledge, no prior studies have examined the desire for touch in psychotherapy. However, this pattern of results aligns with prior findings in romantic relationships, where avoidant attachment negatively correlated with one’s enjoyment of physical affection, such as cuddling with a partner or one’s children (Chopik et al., 2014). Similarly, Carmichael et al. (2021) found that anxious attachment was positively associated with touch desire, while avoidant attachment showed the opposite pattern (Carmichael et al., 2021).

While surveys of psychotherapists suggest that most have used touch at least once in their practice (Pope et al., 1987; Strozier et al., 2003), our findings indicate that most clients did not recall being touched. This discrepancy may reflect differences in recall or that therapists typically work with multiple clients. Future research could explore this potential asymmetry more directly by comparing matched reports from therapists and their clients, although this would need to be conducted with careful ethical considerations. As expected, touch was more common in body-oriented therapies, which is consistent with their focus on the somatic domain and touch as a therapeutic intervention (Hunter & Struve, 1998; Levine & Frederick, 1997; Perls, 1973; Smith et al., 1998).

The finding that most individuals who underwent talking psychotherapy reported never experiencing therapist touch is particularly intriguing, given that 40% of respondents expressed a desire for touch. This discrepancy highlights a potential gap between client preferences and therapeutic practices. While documenting patient desires alone cannot drive changes in therapeutic practice, these findings supports calls to reconsider the integration of embodied approaches into talking psychotherapy treatments, such as CBT (Gennaro et al., 2019). However, it is crucial to recognize that a desire for touch does not imply that such desires should necessarily be acted upon. Therapists must approach any consideration of touch intervention with caution, mindful of ethical and professional guidelines (Zur & Nordmarken, 2011). Moreover, future studies are needed to replicate and expand these findings, as this survey represents an initial exploration of clients’ attitudes toward touch in psychotherapy.

Regarding our second hypothesis on the affective quality of touch, the finding that avoidant attachment scores were negatively associated with positive ratings of touch experiences, while anxious attachment scores were not, aligns with established attachment theory (Ainsworth, 1989). Avoidant individuals tend to distance themselves from intimacy, which likely diminishes their ability to perceive touch experiences as positive. In contrast, anxious individuals’ preoccupation with fears of rejection and ambiguity in addressing intimacy (Ainsworth, 1989) might constrain their capacity to evaluate the affective quality of touch consistently. General attitudes toward touch were associated with more positive perceptions, but contrary to our expectations, developmental touch history was not. The null effect of developmental touch history (i.e., participants’ retrospective accounts of positive physical affection received in childhood, such as hugging, cuddling, and bedtime routines) may stem from the measure’s focus solely on affectionate touch, without accounting for abusive or harmful touch experiences. Research suggests that childhood abuse can hinder individuals’ beliefs about touch, particularly fostering the perception that touch, even in a psychotherapeutic context, is unsafe (Wilson, 1982). Future studies should explore the impact of childhood trauma and adverse touch experiences on attitudes toward therapeutic touch, providing a more comprehensive understanding of these dynamics.

Most individuals who experienced touch in therapy perceived it as supportive and enhancing the therapeutic alliance. These findings align with Horton et al.’s (1995) thematic analysis, where touch was described as fostering closeness and trust (Horton et al., 1995). Consistent with our expectations these perceptions were also associated with general touch attitudes and attachment styles, emphasizing the subjectivity of touch experiences and their variability across clients (Phelan, 2009). However, contrary to our hypothesis, developmental touch history was not associated with these perceptions. Using a different measure of developmental touch history—one that captures both positive and negative/traumatic developmental experiences— may provide a more comprehensive understanding of these relationships.

Importantly, 4% of our sample (*n* = 61) reported experiencing touch they deemed inappropriate. This finding underscores the urgent need for further research—both qualitative and quantitative —alongside enhanced regulatory frameworks and policies aimed at minimizing and ultimately eliminating such risks in psychotherapy. While therapeutic touch appeared to have positive effects for many clients, its potential to cause harm if perceived as inappropriate or intrusive highlights the need for cautious and ethical implementation in practice.

Notably, the use of composite measures, such as “acceptance or support,” or “violated and inappropriate” to measure the overall affective quality of touch experiences, may have inadvertently conflated distinct constructs. Some participants may have viewed touch as symbolically supportive without perceiving it as alliance-enhancing, or vice versa. Similarly, “inappropriate” may not always reflect boundary violation. Future work should disaggregate these constructs using more precise tools.

This study has several limitations that need acknowledgment. Firstly, as a self-report on-line survey, we could not verify participants’ understanding of the questions, particularly those related to the type of psychotherapy they received. To address potential inaccuracies, including those arising from the non-mutually exclusive categories used in the survey, we aggregated therapy types under the broader category of talking therapies, as detailed in the Methods section. Future research should explore distinctions between specific types of psychotherapy, such as CBT and psychodynamic therapy (see supplementary materials).

Secondly, we did not account for other factors that might influence touch desire or experiences, such as childhood adversity (Bernstein et al., 1994), epistemic trust (Campbell et al., 2021), or current mental-health symptoms. Thirdly, our survey employed broad, general questions about touch experiences, often used only one item potentially limiting psychometric reliability. It lacked details on specific touch types (e.g., hugs, handshakes, hand-holding, stroking) or their intensity, which could provide deeper insights into individual client experiences. Additionally, the survey’s design did not allow for comparisons between current and past touch experiences, nor did it clarify whether participants were reflecting on experiences with a single therapist or multiple therapists across different therapeutic contexts. While including more detailed questions would have provided richer data, this would have significantly lengthened the survey. Since the meaning of touch is shaped by context and subjective experience, we prioritized items that assessed perceived meaning over mechanical features (Longa et al., 2022). Notably, a recent meta-analysis on touch interventions by Packheiser et al. (2024) found no significant differences in effect sizes across various types of touch, supporting the broad, exploratory approach adopted in our study.

In conclusion, despite the acknowledged limitations of this exploratory survey, it represents the first large-scale investigate into a neglected topic: clients’ perspectives on touch in psychotherapy. It highlights the importance of advancing the field’s understanding of the clinical and ethical considerations surrounding touch, fostering informed, evidence-based guidelines for its appropriate use in therapeutic settings. From our perspective as practicing psychotherapists and chartered psychologists (MT and AF), this paper is not intended to advocate for or against the use of touch interventions in psychotherapy. Instead, our aim is to shed light on an underexplored topic, with the hope that this preliminary survey will inspire further, applied research, perhaps in smaller, more specific samples, or using Randomized Controlled Trial designs to explore the perception, feasibility and effectiveness of touch interventions. Clinicians should carefully evaluate the therapeutic implications of touch interventions, as well as the potential meaning conveyed by choosing not to use touch. As a universal form of human communication, touch - or its absence - can convey significant messages within the therapeutic relationship. Psychotherapists must critically assess whether incorporating touch aligns with the best interests of their clients and their treatment goals. In this regard, it is important that psychotherapists in training are adequately prepared to navigate the complexities of appropriate, non-erotic touch in therapy. While we do not propose specific curriculum changes, we suggest that training programmes consider providing clearer guidelines and opportunities for supervised discussion around the clinical use of touch. This includes recognizing its potential benefits, risks, and how it may be perceived by clients across different cultural, developmental, and therapeutic contexts. We also highlight the need for further research and professional dialogue to inform evidence-based recommendations.

Additionally, we would like to use this platform to remind clinicians and policymakers of the concerning number of individuals who reported inappropriate or abusive touch in therapy. While ethical guidelines provide an essential framework, they may not be sufficient on their own. To ensure responsible practice, periodic training and evaluation of interventions should be incorporated into therapists’ Continuing Professional Development (CPD). As a professional community, we must remain committed to minimizing harmful occurrences and ensuring that touch, when used, is applied ethically and appropriately, with the utmost care for client safety and well-being.

## Supplementary Material

Supplemental data for this article can be accessed at https://doi.org/10.1080/10503307.2025.2534971.

Supplementary Materials

## Figures and Tables

**Table 1:** Sample demographics.

Psychotherapy in the last 10 years	Gender			
	Female	Male	Non-binary/prefer to self-describe	Prefer not to say
No (a)	8632	3268	74	40
Yes (a)	6059	1456	127	39
Yes (b)	5547	1331	–	–
	Ethnicity			
	English/Welsh/Scottish/Northern Irish/British	Irish	Any other white background	Pakistani
No (a)	10458	178	667	12
Yes (a)	6518	162	638	7
Yes (b)	6137	152	589	–
	African	Chinese	Caribbean	Arab
No (a)	11	21	27	6
Yes (a)	14	9	21	3
Yes (b)	–	–	–	–
	Indian	Bangladeshi	Any other ethnic group (grouped)	Mixed (White Asian)
No (a)	49	5	127	50
Yes (a)	34	7	122	49
Yes (b)	–	–	–	–
	Mixed (White Black Caribbean)	Mixed (White Black African)	Any other mixed group	Prefer not to say
No (a)	21	20	9	83
Yes (a)	22	10	6	59
Yes (b)	–	–	–	–
	Is Religious			
	Yes	No	Prefer not to say or NA’s	
No (a)	2970	8210	564	
Yes (a)	1902	5399	380	
Yes (b)	1734	4836	305	
	Sexuality			
	Heterosexual	Gay or Lesbian	Bisexual	Prefer to self-describe (grouped)
No (a)	10739	291	323	235
Yes (a)	6453	344	473	281
Yes (b)	6138	318	422	–
	Prefer not to say			
No (a)	156			
Yes (a)	130			
Yes (b)	–			

Notes: Number of observations. (a) Before exculsion of individuals with demographic characteristics that were under-represented in the sample (e.g., <1% of respondents); *N* = 19425 and (b) After exclusion; *N* = 6878.

## Clinical or Methodological Significance of this Article

This unprecedented study, utilizing a large sample size, provides evidence regarding the presence, significance, and desirability of touch in various psychotherapeutic settings. The findings suggest that while touch is infrequent in psychotherapy, it is more common than previously assumed and desired by approximately 40% of the sample. While most respondents reported positive experiences, some perceived touch as inappropriate or negative. Moreover, these perceptions and the desirability of touch varied based on the respondent’s self-reported adult attachment style and general attitudes toward touch. Although the evidence provided by this study is not sufficient for direct clinical application, it highlights the need for further research on the use, safety and meaning of touch in psychotherapeutic contexts.

## References

[R1] Ainsworth MS (1979). Infant–mother attachment. American Psychologist.

[R2] Ainsworth MS (1989). Attachments beyond infancy. American Psychologist.

[R3] Atzil-Slonim D, Soma CS, Zhang X, Paz A, Imel ZE (2023). Facilitating dyadic synchrony in psychotherapy sessions: Systematic review and meta-analysis. Psychotherapy Research.

[R4] Berendsen P (2017). The intervention of touch in psychotherapy and trauma treatment. Touch in the Helping Professions: Research, Practice, and Ethics.

[R5] Bernstein DP, Fink L, Handelsman L, Foote J, Lovejoy M, Wenzel K, Sapareto E, Ruggiero J (1994). Initial reliability and validity of a new retrospective measure of child abuse and neglect. The American Journal of Psychiatry.

[R6] Bonitz V (2008). Use of physical touch in the ‘talking cure’: A journey to the outskirts of psychotherapy. Psychotherapy: Theory, Research, Practice, Training.

[R7] Bowlby J (1969). Attachment and loss: Attachment; John Bowlby.

[R8] Bowling NC, Vafeiadou A, Hammond C, Banissy MJ (2024). Extraversion and adult attachment dimensions predict attitudes towards social touch. Journal of Research in Personality.

[R9] Brauer J, Xiao Y, Poulain T, Friederici AD, Schirmer A (2016). Frequency of maternal touch predicts resting activity and connectivity of the developing social brain. Cerebral Cortex (New York, NY: 1991).

[R10] Caldwell C (1997). Ethics and techniques for touch in somatic psychotherapy. Getting in Touch: The Guide to New Body-Centered Therapies.

[R11] Campbell C, Tanzer M, Saunders R, Booker T, Allison E, Li E, O’Dowda C, Luyten P, Fonagy P, Doering S (2021). Development and validation of a self-report measure of epistemic trust. PLoS One.

[R12] Carmichael CL, Goldberg MH, Coyle MA (2021). Security-based differences in touch behavior and its relational benefits. Social Psychological and Personality Science.

[R13] Cary KM, Reid TA, PettyJohn ME, Maas MK, McCauley HL (2022). “They are assuming that we are going to accuse them of rape, and we are assuming that they are going to rape us”: A developmental perspective on emerging adults’ consent conversations post #MeToo. Journal of Interpersonal Violence.

[R14] Changaris M, Payne H, Koch S, Tantia J (2019). The Routledge international handbook of embodied perspectives in psychotherapy.

[R15] Chopik WJ, Edelstein RS, van Anders SM, Wardecker BM, Shipman EL, Samples-Steele CR (2014). Too close for comfort? Adult attachment and cuddling in romantic and parent–child relationships. Personality and Individual Differences.

[R16] Davis I, Rovers M, Petrella C, Rovers M, Malette J, Guirguis-Younger M (2017). Touch in the helping professions: Research, practice, and ethics.

[R17] Debrot A, Stellar JE, MacDonald G, Keltner D, Impett EA (2021). Is touch in romantic relationships universally beneficial for psychological well-being? The role of attachment avoidance. Personality & Social Psychology Bulletin.

[R18] Dunbar RIM (2010). The social role of touch in humans and primates: Behavioural function and neurobiological mechanisms. Neuroscience & Biobehavioral Reviews.

[R19] Durana C (1998). The use of touch in psychotherapy: Ethical and clinical guidelines. Psychotherapy: Theory, Research, Practice, Training.

[R20] Eyckmans S (2009). Handle with care: Touch as a therapeutic tool. Gestalt Journal of Australia and New Zealand.

[R21] Fotopoulou A, Tsakiris M (2017). Mentalizing homeostasis: The social origins of interoceptive inference. Neuropsychoanalysis.

[R22] Gabbard GO (2017). Sexual boundary violations in psychoanalysis: A 30-year retrospective. Psychoanalytic Psychology.

[R23] Geib P, Smith EWL, Clance PR, Imes S (1998). Touch in psychotherapy: Theory, research, and practice.

[R24] Gennaro A, Klienbub JR, Mannarini S, Salvatore S, Palmieri A (2019). Training in psychotherapy: A call for embodied and psychophysiological approaches. Research in Psychotherapy.

[R25] Harrison C, Jones RSP, Huws JC (2012). “We’re people who don’t touch”: Exploring clinical psychologists’ perspectives on their use of touch in therapy. Counselling Psychology Quarterly.

[R26] Holroyd JC, Brodsky AM (1977). Psychologists’ attitudes and practices regarding erotic and nonerotic physical contact with patients. American Psychologist.

[R27] Horton JA, Clance PR, Sterk-Elifson C, Emshoff J (1995). Touch in psychotherapy: A survey of patients’ experiences. Psychotherapy: Theory, Research, Practice, Training.

[R28] Hunter M, Struve J (1998). Challenging the taboo: Support for the ethical use of touch in psychotherapy with sexually compulsive/addicted clients. Sexual Addiction & Compulsivity: The Journal of Treatment and Prevention.

[R29] Jaffe AE, Cero I, DiLillo D (2021). The #MeToo movement and perceptions of sexual assault: College students’ recognition of sexual assault experiences over time. Psychology of Violence.

[R30] Jakubiak BK, Feeney BC (2016). A sense of security: Touch promotes state attachment security. Social Psychological and Personality Science.

[R31] Juul S, Poulsen S, Lunn S, Sørensen P, Jakobsen JC, Simonsen S (2019). Short-term versus long-term psychotherapy for adult psychiatric disorders: A protocol for a systematic review with meta-analysis and trial sequential analysis. Systematic Reviews.

[R32] Kempson DA (2000). Effects of intentional touch on complicated grief of bereaved mothers. Omega: Journal of Death and Dying.

[R33] Krahé C, von Mohr M, Gentsch A, Guy L, Vari C, Nolte T, Fotopoulou A (2018). Sensitivity to CT-optimal, affective touch depends on adult attachment style. Scientific Reports.

[R34] Kuhfuß M, Maldei T, Hetmanek A, Baumann N (2021). Somatic experiencing – effectiveness and key factors of a body-oriented trauma therapy: A scoping literature review. European Journal of Psychotraumatology.

[R35] Leder D, Krucoff MW (2008). The touch that heals: The uses and meanings of touch in the clinical encounter. Journal of Alternative and Complementary Medicine (New York, NY).

[R36] Levine PA, Frederick A (1997). Waking the tiger: The innate capacity to transform overwhelming experiences.

[R37] Longa LD, Valori I, Farroni T (2022). Interpersonal affective touch in a virtual world: Feeling the social presence of others to overcome loneliness. Frontiers in Psychology.

[R38] Lowen A (1976). Bioenergetics. The revolutionary therapy that uses the language of the body to heal the problems of the mind.

[R39] Mcparlin Z, Cerritelli F, Friston KJ, Esteves JE (2022). Therapeutic alliance as active inference: The role of therapeutic touch and synchrony. Frontiers in Psychology.

[R40] Mintz EE (1969). On the rationale of touch in psychotherapy. Psychotherapy: Theory, Research & Practice.

[R41] Montagu A (1978). Touching: The human significance of the skin.

[R42] Morrison I (2016). Keep calm and cuddle on: Social touch as a stress buffer. Adaptive Human Behavior and Physiology.

[R43] Myers C, Thompson G, Hughey L, Young JL, Rhon DI, Rentmeester C (2022). An exploration of clinical variables that enhance therapeutic alliance in patients seeking care for musculoskeletal pain: A mixed methods approach. Musculoskeletal Care.

[R44] Packheiser J, Hartmann H, Fredriksen K, Gazzola V, Keysers C, Michon F (2024). A systematic review and multivariate meta-analysis of the physical and mental health benefits of touch interventions. Nature Human Behaviour.

[R45] Peloquin SM (1989). Helping through touch: The embodiment of caring. Journal of Religion and Health.

[R46] Perls F (1973). The Gestalt approach & eye witness to therapy.

[R47] Phelan JE (2009). Exploring the use of touch in the psychotherapeutic setting: A phenomenological review. Psychotherapy (Chicago, Ill).

[R48] Pope KS, Bouhoutsos JC (1986). Sexual intimacy between therapists and patients.

[R49] Pope KS, Tabachnick BG, Keith-Spiegel P (1987). Ethics of practice: The beliefs and behaviors of psychologists as therapists. American Psychologist.

[R50] Reite M, Barnard KE, Brazelton TB (1990). Touch: The foundation of experience: Full revised and expanded proceedings of Johnson & Johnson pediatric round table X.

[R51] Robinson L, Delgadillo J, Kellet S (2019). The dose-response effect in routinely delivered psychological therapies: A systematic review. Psychotherapy Research.

[R52] Rogers CR (1970). Carl Rogers on encounter groups.

[R53] Salzmann-Erikson M, Eriksson H (2005). Encountering touch: A path to affinity in psychiatric care. Issues in Mental Health Nursing.

[R54] Scheinfeld E, Barney K, Gangi K, Nelson EC, Sinardi CC (2021). Filling the void: Grieving and healing during a socially isolating global pandemic. Journal of Social and Personal Relationships.

[R55] Schlesinger HJ, Appelbaum AH (2000). When words are not enough. Psychoanalytic Inquiry.

[R56] Shaver PR, Mikulincer M (2010). New directions in attachment theory and research. Journal of Social and Personal Relationships.

[R57] Shaver PR, Schachner DA, Mikulincer M (2005). Attachment style, excessive reassurance seeking, relationship processes, and depression. Personality and Social Psychology Bulletin.

[R58] Smith EWL, Clance PR, Imes S (1998). Touch in psychotherapy: Theory, research, and practice.

[R59] Stenzel CL, Rupert PA (2004). Psychologists’ use of touch in individual psychotherapy. Psychotherapy: Theory, Research, Practice, Training.

[R60] Stötter A, Mitsche M, Endler PC, Oleksy P, Kamenschek D, Mosgoeller W, Haring C (2013). Mindfulness-based touch therapy and mindfulness practice in persons with moderate depression. Body, Movement and Dance in Psychotherapy.

[R61] Strozier A, Krizek C, Sale K (2003). Touch: Its use in psychotherapy. Journal of Social Work Practice.

[R62] Suvilehto JT, Glerean E, Dunbar RIM, Hari R, Nummenmaa L (2015). Topography of social touching depends on emotional bonds between humans. Proceedings of the National Academy of Sciences.

[R63] Suvilehto JT, Nummenmaa L, Harada T, Dunbar RIM, Hari R, Turner R, Sadato N, Kitada R (2019). Cross-cultural similarity in relationship-specific social touching. Proceedings of the Royal Society B: Biological Sciences.

[R64] Takeuchi MS, Miyaoka H, Tomoda A, Suzuki M, Liu Q, Kitamura T (2010). The effect of interpersonal touch during childhood on adult attachment and depression: A neglected area of family and developmental psychology?. Journal of Child and Family Studies.

[R65] Trotter PD, McGlone F, Reniers RLEP, Deakin JFW (2018). Construction and validation of the touch experiences and attitudes questionnaire (TEAQ): A self-report measure to determine attitudes toward and experiences of positive touch. Journal of Nonverbal Behavior.

[R66] Tschuschke V, Koemeda-Lutz M, von Wyl A, Crameri A, Schulthess P (2020). The impact of patients’ and therapists’ views of the therapeutic alliance on treatment outcome in psychotherapy. The Journal of Nervous and Mental Disease.

[R67] Turp M (2000). Touch, enjoyment and health: In adult life. European Journal of Psychotherapy, Counselling and Health.

[R68] Ward MK, Meade AW (2023). Dealing with careless responding in survey data: Prevention, identification, and recommended best practices. Annual Review of Psychology.

[R69] Wei M, Russell DW, Mallinckrodt B, Vogel DL (2007). The experiences in close relationship scale (ECR)-short form: Reliability, validity, and factor structure. Journal of Personality Assessment.

[R70] Westland G (2011). Physical touch in psychotherapy: Why are we not touching more?. Body, Movement and Dance in Psychotherapy.

[R71] Wilhelm FH, Kochar AS, Roth WT, Gross JJ (2001). Social anxiety and response to touch: Incongruence between self-evaluative and physiological reactions. Biological Psychology.

[R72] Wilson JM (1982). The value of touch in psychotherapy. American Journal of Orthopsychiatry.

[R73] Woods DL, Craven RF, Whitney J (2005). The effect of therapeutic touch on behavioral symptoms of persons with dementia. Alternative Therapies in Health and Medicine.

[R74] Zur O, Zur O (2007). Boundaries in psychotherapy: Ethical and clinical explorations.

[R75] Zur O, Nordmarken N (2011). To touch or not to touch: Exploring the myth of prohibition on touch In psychotherapy and counseling.

