# Supplementary material for: Touch in psychotherapy: Experiences, desires and attitudes in a large population survey
Source: Psychother Res. Author manuscript; Available in PMC 2026 Nov 7. (PMC7619712; doi:10.1080/10503307.2025.2534971)
Supplement: Supplementary file 1 [file EMS220415-supplement-Supplementary_Materials.docx]

**Supplementary Materials**

Table 1S.

*Items, Scales and Frequency Analysis of the Survey (n=6878) in the Different Therapy Groups (*body-oriented’ vs ‘non-body-oriented therapies) *and in Total.*

| Item | Therapist Touch Desire | | | | | | | | | | | | | | |
| --- | --- | --- | --- | --- | --- | --- | --- | --- | --- | --- | --- | --- | --- | --- | --- |
|  | Group Therapy | | Never | | | Almost Never | Rarely | Sometimes | | Often | | Almost always | | Always | NAs |
| Have you ever wanted your therapist to hug, hold or touch you in some way? | Non-body oriented | | 2923  (48%) | | | 901  (15%) | 790  (13%) | 1204  (20%) | | 206  (3%) | | 64  (1%) | | 36  (0.01%) | 5  (0.00%) |
|  | Body-oriented | | 138  (24%) | | | 48  (8%) | 78  (14%) | 203  (37%) | | 58  (10%) | | 23  (4%) | | 19  (3%) | 3  (0.00%) |
|  | Not sure/Prefer not to say | | 53  (30%) | | | 20  (11%) | 16  (9%) | 42  (23%) | | 14  (1%) | | 2  (2.25%) | | 4  (2%) | 28  (16%) |
|  | Total | | 3114  (45%) | | | 969  (14%) | 884  (13%) | 1449  (21%) | | 278  (4%) | | 89  (1%) | | 55  (1%) | 36  (0.05%) |
|  | Amount of physical touch experiences | | | | | | | | | | | | | | |
|  | Group Therapy | | | Never | | | Once | Less than 10 times | | | More than 10 times | | NAs | |  |
| Have you ever experienced some sort of physical contact with your therapist, beyond accidental contact or a formal handshake? | Non-body oriented | | | 4583  (75%) | | | 576  (9%) | 537  (9%) | | | 157  (3%) | | 276  (5%) | |  |
|  | Body-oriented | | | 196  (34%) | | | 39  (7%) | 171  (30%) | | | 159  (28%) | | 5  (1%) | |  |
|  | Not sure/Prefer not to say | | | 100  (56%) | | | 12  (7%) | 25  (14%) | | | 13  (7%) | | 29  (16%) | |  |
|  | Total | | | 4879  (71%) | | | 627  (9%) | 733  (11%) | | | 329  (5%) | | 310  (5%) | |  |
|  | Positive or negative psychotherapy touch experiences* | | | | | | | | | | | | | | |
|  | Group Therapy | Very negative | | | Negative | | Somewhat negative | Neutral | | Somewhat positive | | Positive | | Very positive | NAs |
| Overall, how do you regard the physical contact which occurred in your therapy? | Non-body oriented | 17  (1%) | | | 14  (1%) | | 35  (3%) | 145  (11%) | | 255  (20%) | | 472  (37%) | | 329  (26%) | 4862 |
|  | Body-oriented | 5  (1%) | | | 3  (1%) | | 4  (1%) | 20  (5%) | | 57  (15%) | | 130  (35%) | | 153  (41%) | 198 |
|  | Not sure/  Prefer not to say | 1  (0%) | | | 2  (1%) | | 4  (8%) | 7  (14%) | | 8  (16%) | | 15  (30%) | | 13  (26%) | 129 |
|  | Total | 23  (1%) | | | 19  (1%) | | 43  (3%) | 172  (10%) | | 320  (19%) | | 617  (37%) | | 495  (29%) | 5189 |
| How were the feelings about yourself affected by the touch? | Non-body oriented | 10  (1%) | | | 10  (1%) | | 31  (2%) | 221  (17%) | | 290  (23%) | | 479  (38%) | | 226  (18%) | 4862 |
|  | Body-oriented | 2  (0%) | | | 4  (1%) | | 6  (1%) | 39  (10%) | | 66  (18%) | | 142  (38%) | | 113  (30%) | 198 |
|  | Not sure/  Prefer not to say | 0  (0%) | | | 3  (5%) | | 2  (4%) | 8  (14%) | | 13  (23%) | | 14  (25%) | | 16  (28%) | 129 |
|  | Total | 12  (1%) | | | 17  (1%) | | 39  (2%) | 268  (16%) | | 369  (22%) | | 635  (37%) | | 355  (21%) | 5189 |
| How were your feelings about your therapist affected by the touch? | Non-body oriented | 17  (1%) | | | 8  (1%) | | 35  (3%) | 217  (17%) | | 271  (21%) | | 483  (38%) | | 234  (18%) | 4864 |
|  | Body-oriented | 3  (1%) | | | 6  (2%) | | 5  (1%) | 57  (15%) | | 66  (18%) | | 140  (38%) | | 95  (26%) | 198 |
|  | Not sure/  Prefer not to say | 2  (2%) | | | 1  (2%) | | 4  (8%) | 5  (10%) | | 13  (25%) | | 15  (29%) | | 11  (22%) | 128 |
|  | Total | 22  (1%) | | | 15  (1%) | | 44  (3%) | 279  (16%) | | 350  (21%) | | 638  (38%) | | 340  (20%) | 5190 |
|  |  | Touch supportive or inappropriate | | | | | | | | | | | | | |
|  | Group Therapy | Never | | | Almost never | | Infrequently | | Neutral | Frequently | | Almost every time | | Every time | NAs |
| To what degree did you feel the touch communicated acceptance or support and enhanced your connection, or bond with the therapist? | Non-body oriented | 41  (3%) | | | 19  (1%) | | 15  (1%) | | 150  (12%) | 228  (18%) | | 189  (15%) | | 624  (49%) | 4863 |
|  | Body-oriented | 8  (2%) | | | 8  (2%) | | 0  (0%) | | 29  (8%) | 60  (16%) | | 82  (22%) | | 185  (50%) | 198 |
|  | Not sure/  Prefer not to say | 2  (4%) | | | 1  (2%) | | 2  (4%) | | 10  (19%) | 10  (19%) | | 7  (14%) | | 19  (37%) | 129 |
|  | Total | 51  (3%) | | | 28  (1%) | | 16  (0%) | | 189  (12%) | 298  (18%) | | 278  (16%) | | 828  (49%) | 5190 |
| To what degree did you feel the touch violated the boundaries of the therapeutic relationship and was inappropriate? | Non-body oriented | 988  (78%) | | | 122  (10%) | | 36  (3%) | | 70  (6%) | 20  (2%) | | 6  (0%) | | 25  (2%) | 4862 |
|  | Body-oriented | 297  (80%) | | | 42  (11%) | | 8  (0%) | | 17  (5%) | 2  (0%) | | 4  (0%) | | 2  (0% | 198 |
|  | Not sure/  Prefer not to say | 36  (72%) | | | 4  (8%) | | 4  (8%) | | 4  (8%) | 1  (2%) | | 1  (2%) | | 0  (0%) | 129 |
|  | Total | 1321  (78%) | | | 168  (10%) | | 48  (3%) | | 91  (5%) | 23  (1%) | | 11  (1%) | | 27  (2%) | 5189 |

**Note*. Overall ratings of touch experiences were assessed using an average score of the three items.

**Figure 1** Age histogram showing a disproportionate spike in participants reporting their age as 18 (n = 147), compared to adjacent age groups (e.g., ages 19–21: n = 157 combined).

**Additional Measures**

Below we present analyses using additional measures (e.g., Empathy, Touch-loneliness) and comparisons of CBT vs non-CBT. All these additional measures and hypotheses were pre-registered (see link). However, we report these findings as supplementary information to focus on the main results and because we could not fully verify reliability of self-reports regarding CBT (see also p.13 main text).

For analyses relating to CBT therapy vs non-CBT, we aggregated psychotherapy, counselling and talking therapy as one ‘non-CBT talking therapy’ category. For responses marked as ‘other’ (n=340), we classified them, when possible and based on the evaluation of three practicing psychologists we classified them into either as ‘non-CBT talking therapies’ (i.e., ’Psychoanalysis’, ‘Grief and bereavement counselling’, ‘family therapy’ etc), or ‘CBT’ (i.e., ‘EMDR’, ‘ACT’, ‘DBT’ etc) or ‘Other’ (‘homeopathy’, ‘healer’, ‘hypnosis’ etc). Specifically, two researchers (MT and IG), both practicing psychologists with relevant expertise and identical, a priori training based on surveying together the websites of therapeutic and psychological associations, societies and organisations in the UK, independently reviewed these responses. In cases of disagreement, a third researcher (AF), also a practicing, chartered psychologist with extensive clinical and research experience in the area, was consulted to reach a consensus.

**Instruments**

*Empathy*

Empathy scores were assessed using the 5-items cognitive empathy subscale from the Empathy Quotient-Short Form (EQ-15; (Muncer & Ling, 2006). Items were rated on a 4-scale from “Strongly disagree” to “Strongly agree”. The EQ-15 assess empathy in adults and shows excellent reliability and validity (Paolo Senese et al., 2018). For this study, Cronbach's α = .84.

*Touch-Loneliness.*

Feelings of loneliness related to touch were measured with a single item: “When/if your social relationships do not include touch, do you notice a difference in how lonely you feel? (von Mohr et al., 2021)). Responses were recorded on a 5-scale from “Never” to “Always”. Higher scores reflect greater loneliness in response to the absence touch in social relationships.

**Results**

**Touch Desire in Relation to Therapy group (CBT vs non-CBT), Duration, Attachment and Touch Attitudes.**

To explore touch-interventions within the different psychotherapy modalities, we hypothesised that individuals who have had CBT, given its goal-oriented, directive, and symptom- focused approach, would report less desire for touch in therapy compared to those in the non-CBT modalities. To test this, we conducted a separate analysis (R_Marginal_^2^ = 0.03; n=6768) comparing individuals who have had CBT with those receiving non-CBT therapies. As hypothesised, participants in CBT group reported a significantly lower desire for touch (*OR* = 0.83, *p* =.001, *95%CI* [0.73;0.93]).

Furthermore, attachment style dimensions influenced the desire for touch within the CBT group. Higher anxious attachment scores were associated with an increased desire for touch (*OR* = 1.26 *p* <.001, *95%CI* [1.18;1.35]), while higher avoidant attachment scores were linked to a decreased desire for touch (*OR* = 0.89 *p* =.002, *95%CI* [0.83;0.96]).

**Affective quality of touch experiences in relation to developmental touch history, attachment, attitudes to touch and therapy type.**

Using a multilevel modelling (MLM), we examined the continuous dependent variable *affective quality of touch experiences*, with the categorical variable ‘Therapy group’ (comprising of ‘Body-oriented’, ‘non-CBT’ or ‘CBT’), and continuous predictors including attachment style dimension, attitudes toward touch, touch loneliness feelings and empathy scores (R_Marginal_^2^ = 0.10; n=1610). We found no evidence that the categorical variable ‘Therapy group’ was associated with the affective quality of touch experiences. As predicted (see pre-registration), touch loneliness feelings were positively associated with the affective quality of touch experiences (*b* = 0.12, *p* <.001, *95%CI* [0.07;0.18]), indicating that greater loneliness feelings corresponded to more positive therapeutic touch experiences. Empathy scores were not significantly associated with the affective quality of touch experiences (*b* = 0.02, *p* =.52, *95%CI* [-0.04;0.07]).

Table 2S. *Summary of Pearson’s intercorrelations of independent variables.*

|  | 1 | 2 | 3 | 4 | 5 | 6 | 7 | 8 | 9 | 10 |
| --- | --- | --- | --- | --- | --- | --- | --- | --- | --- | --- |
| 1. Att-anx | --- | 0.11 | -0.13 | -0.07 | -0.08 | -0.19 | -0.02 | 0.08 | 0.3 | -0.06 |
| 2. Att-avoid |  | --- | -0.24 | -0.23 | -0.38 | -0.39 | -0.3 | -0.13 | -0.03 | -0.14 |
| 3. Dis phys |  |  | --- | 0.16` | 0.33 | 0.14 | 0.63 | 0.18 | 0.19 | 0.15 |
| 4. Dev hist |  |  |  | --- | 0.17 | 0.24 | 0.28 | 0.15 | 0.03 | 0.09 |
| 5. Att int |  |  |  |  | --- | 0.29 | 0.43 | 0.24 | 0.25 | 0.09 |
| 6. Cur int |  |  |  |  |  | --- | 0.26 | 0.13 | -0.10 | 0.13 |
| 7. Lik phys |  |  |  |  |  |  | --- | 0.34 | 0.31 | 0.26 |
| 8. Att self-care |  |  |  |  |  |  |  | --- | 0.15 | 0.20 |
| 9. Touch lon |  |  |  |  |  |  |  |  | --- | 0.08 |
| 10. Empathy |  |  |  |  |  |  |  |  |  | --- |

*Note.* Att-anx = Attachment-anxiety; Att-avoid = Attachment-avoidance; TEAQ_STQ factors: Dis phys = Disliking of physical touch; Dev hist = Developmental touch history; Att int = Attitudes to intimate touch; Cur int = Current intimate touch; Lik phys = Liking of physical touch; Att self-care = Attitudes to self-care; Touch lon = Touch-loneliness.

Table 3S.

*MLM analysis of attachment style, positive touch attitudes on the desire for touch, controlling for age, gender, sexuality, ethnicity and religion (R_Marginal_^2^ = 0.013; n=4816).*

| Predictors | b | 95% CI | *p* |
| --- | --- | --- | --- |
| Age | -0.01 | -0.00;0.01 | <.001 |
| Attachment-anxiety | 0.19 | 0.15;0.22 | <.001 |
| Attachment-avoidance | 0.01 | -0.03; 0.04 | .71 |
| TEAQ_STQ total: | 0.38 | 0.34;0.41 | <.001 |
| Therapy duration | 0.47 | 0.41;-0.54 | <.001 |

Table 4S.

*MLM analysis of attachment style, touch attitudes, psychotherapy modalities, touch loneliness and empathy scores on the affective quality of touch experiences, controlling for the amount of touch, age, gender, sexuality, ethnicity and religion (R_Marginal_^2^ = 0.010 for all models; n=1610 and n=1597 for model including Empathy/Loneliness).*

| Predictors | b | 95% CI | *p* |
| --- | --- | --- | --- |
| Age | -0.00 | -0.00;0.00 | 0.87 |
| Attachment-anxiety | -0.03 | -0.08;0.02 | 0.28 |
| Attachment-avoidance | -0.11 | -0.16;-0.06 | <0.001 |
| TEAQ_STQ factors: |  |  |  |
| Disliking of physical touch | -0.23 | -0.29;-0.18 | <0.001 |
| Developmental touch history | -0.01 | -0.06;0.04 | 0.61 |
| Attitudes to intimate touch | 0.11 | 0.06;0.16 | <0.001 |
| Current intimate touch | -0.01 | -0.07;0.04 | 0.58 |
| Liking of physical touch | 0.25 | 0.19;0.31 | <0.001 |
| Attitudes to self-care | 0.08 | 0.03;0.14 | 0.004 |
| Psychotherapy modality: |  |  |  |
| a) Non-CBT | 0.00 | -0.23;0.23 | 0.99 |
| b) CBT | -0.02 | -0.16;0.12 | 0.78 |
| c) Body-oriented therapy | 0.06 | -0.20;0.32 | 0.67 |
| Touch-loneliness | 0.12 | 0.07;0.18 | <0.001 |
| Empathy | 0.02 | -0.04;0.07 | 0.52 |

Table 5S

*Linear multiple regression of attachment style, touch attitudes and psychotherapy modalities on the of touch experiences in therapy to perceived as (A) inappropriate (R^2^_Marginal_=0.04; n=1610) or (B) supportive (R^2^_Marginal_=0.05; n=1609), controlling for the amount of touch, age, gender, sexuality, ethnicity and religion.*

| A. **Dependent variable:** Touch perceived as inappropriate | | | |
| --- | --- | --- | --- |
| predictors | b | 95% CI | p |
| Age | 0.00 | -0.00;0.01 | 0.83 |
| Attachment-anxiety | 0.10 | 0.04;0.15 | 0.001 |
| Attachment-avoidance | 0.06 | -0.00;0.11 | 0.056 |
| TEAQ_STQ factors: |  |  |  |
| Disliking of physical touch | 0.17 | 0.11;-0.23 | <0.001 |
| Developmental touch history | -0.00 | -0.06;0.06 | 0.93 |
| Attitudes to intimate touch | -0.02 | -0.08;0.04 | 0.48 |
| Current intimate touch | 0.01 | -0.05;0.07 | 0.66 |
| Liking of physical touch | -0.12 | -0.18;-0.06 | <0.001 |
| Attitudes to self-care | -0.02 | -0.08;0.04 | 0.47 |
| **Dependent variable:** Touch perceived as supportive | | | |
| predictors | b | 95% CI | p |
| Age | -0.00 | -0.01;0.01 | 0.97 |
| Attachment-anxiety | -0.01 | -0.08;0.06 | 0.75 |
| Attachment-avoidance | -0.10 | -0.17;-0.03 | 0.007 |
| TEAQ_STQ factors: |  |  |  |
| Disliking of physical touch | -0.19 | -0.26;-0.11 | <0.001 |
| Developmental touch history | -0.00 | -0.07;0.07 | 0.99 |
| Attitudes to intimate touch | 0.11 | 0.04;0.17 | 0.003 |
| Current intimate touch | -0.01 | -0.07;0.08 | 0.89 |
| Liking of physical touch | 0.23 | 0.16;0.30 | <0.001 |

**Exploratory analyses: Attachment styles and body-oriented therapy**

To examine whether participants with different attachment styles (anxious or avoidant) were more likely to engage in body-oriented therapy we conducted exploratory analyses. We found no statistically significant associations between attachment style and engagement in body-oriented therapy. Specifically, logistic regression models indicated that neither anxious attachment (OR = -0.08, p = .19, 95% CI [-0.20, 0.04]) nor avoidant attachment (OR = -0.08, p = .13, 95% CI [-0.19, 0.02]) was significantly related to the odds of clients participating in a therapy that involved body-oriented elements.

**References**

Muncer, S. J., & Ling, J. (2006). Psychometric analysis of the empathy quotient (EQ) scale. *Personality and Individual Differences*, *40*(6), 1111–1119. <https://doi.org/10.1016/j.paid.2011.07.005>

Trotter, P.D., McGlone, F., Reniers, L.E.P., & Deakin, J.F.W. (2018). Construction and validation of the touch experiences and attitudes questionnaire (TEAQ): A Self-report measure to determine attitudes toward and experiences of positive touch. *Journal of Nonverbal Behavior*, *42*(4), 379–416. https://doi.org/10.1007/s10919-018-0281-8

von Mohr, M., Kirsch, L. P., & Fotopoulou, A. (2021). Social touch deprivation during COVID-19: Effects on psychological wellbeing and craving interpersonal touch. *Royal Society Open Science*, *8*(9), 210287. https://doi.org/10.1098/rsos.210287

Wilhelm, F. H., Kochar, A. S., Roth, W. T., & Gross, J. J. (2001). Social anxiety and response to touch: Incongruence between self-evaluative and physiological reactions. *Biological Psychology*, *58*(3), 181–202. https://doi.org/10.1016/S0301-0511(01)00113-2
